# Alginate Oligosaccharides in Lipid Metabolism: A Dual-Action Marine Prebiotic

**DOI:** 10.3390/nu18183093

**Published:** 2026-09-21

**Authors:** Jiaqi Liang, Ruibo Zhang, Meiling Song, Wanghua Bi, Quancai Li, Zhen Chen

**Affiliations:** 1 School of Pharmacy, Jiangsu University, Zhenjiang 212013, China; liangjiaqi@stmail.ujs.edu.cn (J.L.); zhangruibo@stmail.ujs.edu.cn (R.Z.); songmeiling@stmail.ujs.edu.cn (M.S.); 2 Marine Biomedical Research Institute of Qingdao, Qingdao 266071, China; sw2005ben3@163.com; 3 Shandong Key Laboratory of Glycoscience and Glycotherapeutics, Ocean University of China, Qingdao 266003, China

**Keywords:** charged oligosaccharides, prebiotics, gut microbiota, short-chain fatty acids, metabolic disorders, metabolic dysfunction-associated steatotic liver disease, dyslipidemia

## Abstract

Alginate oligosaccharides (AOS) are degradation products of brown seaweed alginate, known as dietary bioactive components and used as health-beneficial nutrients. Unlike conventional prebiotics such as fructo-oligosaccharides, galacto-oligosaccharides, and xylo-oligosaccharides, AOS carry carboxyl groups and are negatively charged at physiological pH. This narrative review uses charge as an organizing framework for understanding their lipid-regulating effects. Neutral oligosaccharides act mainly through gut microbial fermentation, whereas charged oligosaccharides can also act on host cells directly, although charge and backbone structure cannot be fully separated in the available studies. The gut-dependent actions of AOS (microbiota modulation, short-chain fatty acid production, bile acid metabolism, and barrier protection) and the direct actions supported mainly by in vitro evidence or parenteral administration in animals, including AMP-activated protein kinase (AMPK) activation, brown adipose tissue thermogenesis, and candidate receptor-mediated effects, are discussed, although the systemic exposure achievable after oral intake remains to be established. The beneficial functions of AOS depend on structural parameters such as degree of polymerization, M/G ratio, and terminal unsaturation; the AOS product approved in China in 2026 is produced by acid hydrolysis, and its relationship to the more active unsaturated forms remains unresolved. Structurally defined AOS are to be developed for nutritional supplementation and as potential candidates for clinical evaluation.

## 1. Introduction

Lipid metabolism disorders, including dyslipidemia and metabolic dysfunction-associated steatotic liver disease (MASLD; formerly termed non-alcoholic fatty liver disease, NAFLD), are common worldwide and contribute to cardiovascular disease [1,2]. The prevalence of MASLD alone exceeds 30% globally, making diet an important target for intervention [1]. Dietary intervention is an established strategy for managing these conditions [3], and among the dietary strategies, prebiotic oligosaccharides are studied for their metabolic benefits.

**Figure 1:**
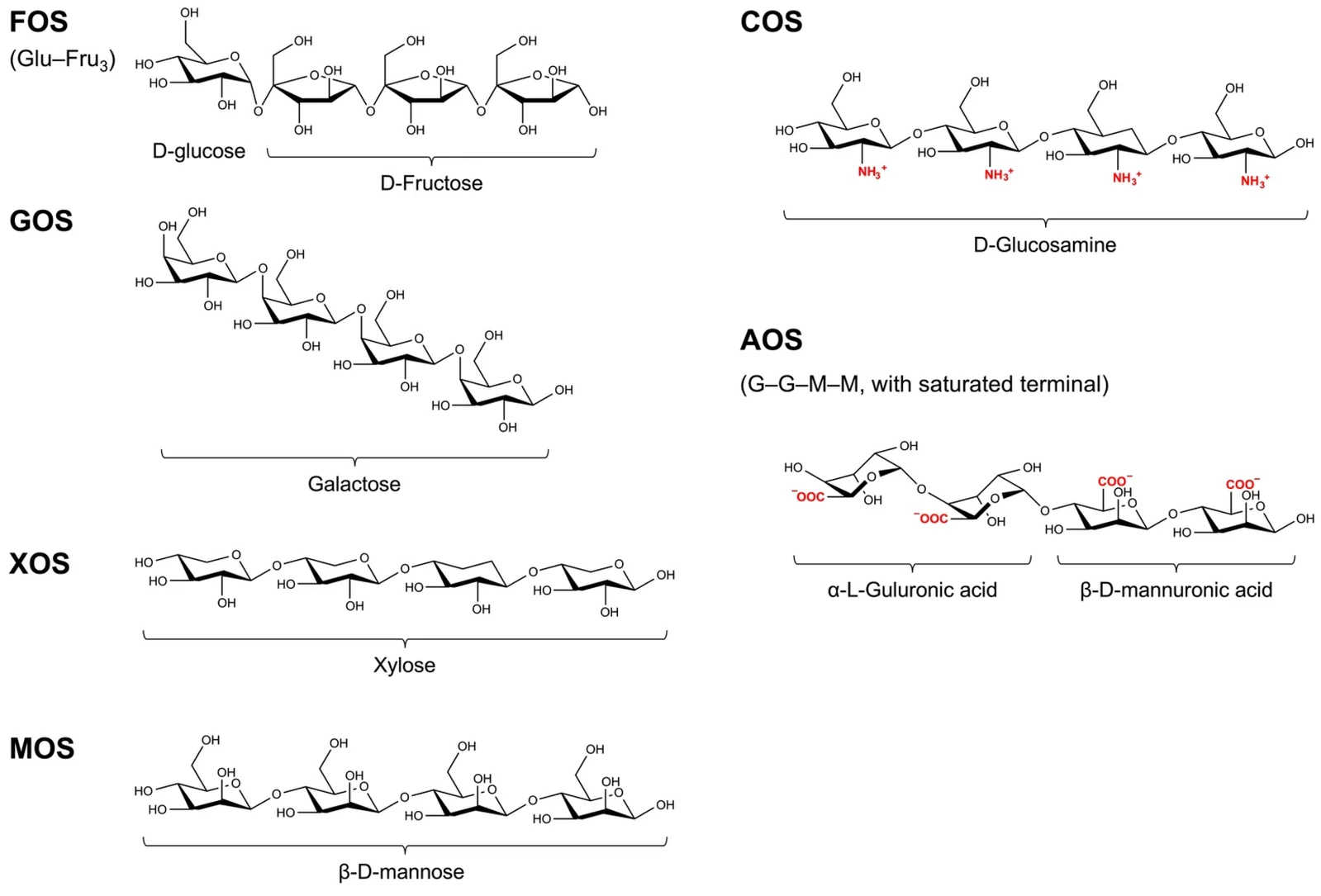
Representative chemical structures of the neutral oligosaccharides FOS, GOS, XOS, and MOS and the charged oligosaccharides COS and AOS. Red highlights the ionizable groups responsible for the charge difference.

Not all oligosaccharides work through the same mechanisms. Conventional prebiotic oligosaccharides, such as fructo-oligosaccharides (FOS), galacto-oligosaccharides (GOS), xylo-oligosaccharides (XOS), and manno-oligosaccharides (MOS) are neutral carbohydrates, containing only hydroxyl groups and no ionizable groups (Figure 1). They act through gut microbial fermentation and short-chain fatty acid (SCFA) production, which contribute to anti-inflammatory, antioxidant, and metabolism-regulating actions, maintaining gut and body health [4,5,6]. Besides, some neutral oligosaccharides have also been reported to exert direct (i.e., microbiota-independent) effects on intestinal barrier function, although fermentation remains their primary mechanism of action [7].

Certain oligosaccharides carry ionizable groups and are chemically different, among which alginate oligosaccharides (AOS) and chitosan oligosaccharides (COS) are the most studied examples (Figure 1): AOS are anionic (negatively charged) while COS are cationic (positively charged). The negative charge at physiological pH, derived from carboxylate groups on their uronic acid residues [8,9], is proposed to enable direct host–cell interactions, although direct evidence isolating charge from backbone structure is still limited. AOS may bind directly to host cell receptors such as the candidate mannose receptor C-type 1 (MRC1) [10], in addition to undergoing conventional gut-dependent fermentation [11,12]. This dual mode of action, combining microbiota-independent host–cell interactions with conventional gut-dependent fermentation, distinguishes AOS from neutral prebiotics, which rely only on fermentation. AOS were recently approved as a novel food raw material in China (National Health Commission, 2026) [13,14], although human data on lipid outcomes are still lacking.

Charge cannot be fully separated from monomer composition, glycosidic linkage, or molecular weight in the available studies, so any charge-based framework should be regarded as a heuristic for generating testable hypotheses rather than as a claim of exclusive causality. However, despite these limits, a systematic conceptual framework linking charge polarity to oligosaccharide mechanisms is still lacking. While direct effects of non-digestible oligosaccharides on intestinal barrier function have been reported [7], the role of charge polarity as an important structural factor of these effects has not been systematically examined. Therefore, the present review proposes a charge-based classification of oligosaccharides as such an organizing framework and examines how it explains the divergent mechanisms of AOS versus neutral prebiotics in lipid metabolism regulation. Current evidence, methodological limitations, and future directions are discussed.

## 2. Materials and Methods

### 2.1. Literature Search Strategy

A comprehensive literature search was conducted to identify studies on AOS, their effects on lipid metabolism, and their mechanisms of action. Electronic searches were performed in PubMed, Web of Science, and Scopus. The search strategy combined terms related to alginate oligosaccharides (e.g., “alginate oligosaccharide”, “alginate oligomer”, “alginate-derived oligosaccharide”, “AOS”, “oligomannuronate”, “oligoguluronate”, “brown algae oligosaccharide”) with terms related to lipid metabolism (e.g., “lipid metabolism”, “lipogenesis”, “fatty acid oxidation”, “cholesterol”, “triglyceride”, “obesity”, “steatosis”, “NAFLD”, “MASLD”), gut microbiota (e.g., “gut microbiota”, “prebiotic”, “short-chain fatty acid”, “bile acid”, “intestinal barrier”), and related oligosaccharide classes used for comparison (e.g., “fructo-oligosaccharide”, “galacto-oligosaccharide”, “xylo-oligosaccharide”, “manno-oligosaccharide”, “chitosan oligosaccharide”, “charged oligosaccharide”). The search was limited to English-language publications in the databases listed above, with no date restriction, and the last update was performed on 31 July 2026.

To cover the industrial and regulatory landscape, official regulatory documents (e.g., announcements of the National Health Commission of China, which are published in Chinese), as well as market research reports were searched separately, with key terms translated into Chinese by the authors where necessary. In addition, the reference lists of retrieved articles were manually screened to identify further relevant studies.

### 2.2. Study Selection and Eligibility Criteria

After removing duplicates, titles and abstracts were screened for relevance to AOS and lipid metabolism. Full-text articles were then assessed against predefined eligibility criteria. Studies were included if they: (1) investigated the effects of alginate oligosaccharides or structurally related charged oligosaccharides (e.g., chitosan oligosaccharides) on lipid metabolism in any experimental model (in vitro, in vivo, or clinical); (2) addressed the gut-dependent or gut-independent mechanisms of oligosaccharide bioactivity; (3) provided data on the structural characterization, production, or regulatory status of AOS; or (4) offered comparative information on neutral or charged oligosaccharides relevant to the charge-based classification framework. Both original research articles and reviews were considered. Studies were excluded if they were: (1) conference abstracts, patents, or commentaries; (2) not available in full text; or (3) primarily focused on alginate polymers or other polysaccharides without data on oligosaccharide-specific effects.

## 3. Oligosaccharide Classification by Charge: A Functional Framework

### 3.1. Neutral Oligosaccharides Rely Primarily on Microbiota-Dependent Fermentation to SCFAs

Neutral oligosaccharides (FOS, GOS, XOS, MOS) share a defining chemical feature: they contain no ionizable functional groups at physiological pH. This single property has important biological consequences. The most extensively characterized mechanism of neutral oligosaccharides is their fermentation by gut microbes to SCFAs. The SCFA signaling cascade has been elucidated at high resolution: cryo-electron microscopy structures of free fatty acid receptors 2 and 3 (FFAR2, FFAR3; also known as GPR43, GPR41) reveal how SCFAs are recognized and activate these receptors [15]. Downstream, SCFAs activate GPR41/GPR43–extracellular signal-regulated kinase 1/2 (ERK1/2)–p38 mitogen-activated protein kinase (MAPK) signaling in intestinal epithelial cells and inhibit histone deacetylases (HDACs) in immune cells [4]. Fructo-oligosaccharides (FOS, DP 2–10), also known as oligofructose and consisting of fructose and glucose units, are produced from chicory inulin or synthesized from sucrose [16,17]; galacto-oligosaccharides (GOS, DP 2–8), derived from lactose, are galactose-based oligomers that selectively promote *Bifidobacterium* proliferation [18]. Xylo-oligosaccharides (XOS, DP 2–10) are derived from lignocellulosic biomass and are fermented by bifidobacteria and lactobacilli [19,20]. Manno-oligosaccharides (MOS) from yeast cell walls have documented prebiotic and immunomodulatory effects [21]. Despite structural differences in source, composition, and chain length, conventional neutral prebiotics (FOS, GOS, XOS, MOS) share a common mechanistic feature, i.e., microbiota-dependent fermentation to SCFAs [19,21,22]. Some structurally complex neutral oligosaccharides, particularly human milk oligosaccharides, have been reported to engage in direct immune receptor interactions through lectin-mediated recognition, indicating that structural features beyond charge may also enable direct host interactions under certain conditions [23]. Animal experiments provide direct evidence for this fermentation-dependent mechanism. In high-fat diet models, inulin and cellulose improve metabolic parameters, and these effects are eliminated when the gut microbiota is depleted by antibiotics; for other neutral oligosaccharides such as FOS, GOS, XOS, and MOS, antibiotic-depletion evidence is more limited, and generalization of this finding to the whole class should be made with caution. Radiolabeled FOS is not metabolized in germ-free or antibiotic-treated rats, confirming that host enzymes cannot directly break down these oligosaccharides [24]. Fecal microbiota transplantation (FMT) from inulin-fed animals transfers metabolic benefits to recipient mice through an IL-22-dependent mechanism, confirming the causal role of the microbiota in that model; comparable FMT data for other neutral oligosaccharides are not yet available [25].

Clinical studies provide complementary evidence at the population level. A meta-analysis of 55 randomized controlled trials found that inulin-type fructans reduce low-density lipoprotein (LDL) cholesterol and body weight, but the effect sizes are modest (LDL reduction of approximately 5%) and show high inter-study heterogeneity [26]. Several trials have reported negative or marginal results: FOS at 20 g/day showed no effect on glucose or lipid metabolism in type 2 diabetes patients [27], and short-chain FOS failed to meet its primary HbA1c endpoint in overweight prediabetic adults [28]. FOS and GOS supplementation in healthy young adults increased *Bifidobacterium* but unexpectedly reduced butyrate-producing bacteria, correlating with adverse glycemic changes [29].

The source of this variability lies in the host microbiota itself. Individual responses to different prebiotics are highly correlated within the same person, and baseline short-chain fatty acid concentrations (determined by habitual fiber intake) predict the magnitude of response [30]. The ratio of Bacteroidetes to *Bifidobacterium* at baseline predicts the gut microbiota response more strongly than the type of prebiotic administered [31]. Even at low doses (1.3 g/day), GOS increase *Bifidobacterium*, but responders differ from non-responders in their baseline microbiota composition [32].

### 3.2. Charged Oligosaccharides: A Charge-Based Framework for Direct Host Interactions and Luminal Actions

Charged oligosaccharides carry ionizable groups that dissociate at physiological pH. It should be emphasized that charge polarity cannot be experimentally separated from backbone structure (monomer composition, glycosidic linkage, and chain length) in the available studies; the charge-based framework is therefore proposed as a heuristic for predicting mode of action rather than as evidence that charge alone drives biological activity. This chemical feature enables oligosaccharides to engage in direct electrostatic interactions with host cells, bacterial surfaces, and luminal components [9,33]. AOS and COS are both charged, but their opposite polarities direct them to different targets. The negative charge of AOS favors interactions with host receptors and bacterial surfaces, producing direct signaling and barrier effects. The positive charge of COS favors interactions with negatively charged molecules in the lumen, mainly affecting nutrient absorption. This contrast is visible in the same Caco-2 model, where AOS protected the barrier against toxin challenge while COS showed stronger antibacterial activity [34]. Direct signaling pathways have been reported for AOS, though their downstream details remain incompletely characterized, and no human lipid data are available, while COS have human trial data but a luminal mode of action. Charge polarity thus offers a heuristic for predicting the mode of action of an oligosaccharide. This framework also has practical implications. Individual variation in the gut microbiota strongly shapes the response to fermentation-dependent interventions [35], whereas the direct mechanisms of charged oligosaccharides are less dependent on the host microbiota.

#### 3.2.1. Anionic AOS via Direct Interactions with Host Cells

AOS carry a negative charge because the carboxylate groups (-COO-) of their uronic acid residues ionize at physiological pH (pKa 3.4 to 4.4) [36]. This anionic character enables direct interactions with host receptors. Molecular docking and knockout studies suggested MRC1 (CD206) as a candidate mediator of AOS cytoprotection in renal ischemia–reperfusion injury, and the protective effect was eliminated in MRC1-knockout mice [10]. Also, AOS suppress toll-like receptor 4 (TLR4)/myeloid differentiation primary response 88 (MyD88)/nuclear factor kappa B (NF-κB) signaling and upregulate tight junction proteins in lipopolysaccharide (LPS)-challenged intestinal epithelium [37]. The negative charge further allows electrostatic interactions with bacterial surfaces. G-block-enriched AOS bind *Pseudomonas aeruginosa* lipopolysaccharide and interfere with quorum sensing [33].

Whether these direct interactions require the gut microbiota is debated. Li et al. found that unsaturated AOS improved metabolic abnormalities in high-fat diet-fed mice, but antibiotic treatment reversed these effects, suggesting a microbiota-dependent component [12]. In contrast, Xu et al. showed that orally administered AOS alleviated obesity by promoting brown adipose tissue thermogenesis, an effect that persisted after antibiotic depletion of the gut microbiota [38]. The discrepancy may reflect differences in the AOS preparations, doses, and experimental conditions. The bioactivities of AOS may be attributed to the structural features like terminal 4,5-unsaturation and/or a low degree of polymerization. For example, the antibiotic-reversible effects were obtained with unsaturated AOS (UAOS), whereas the antibiotic-resistant effects were obtained with a low-DP AOS preparation [38]. This divergence is itself consistent with the charge-based framework developed here, which predicts that terminal unsaturation shifts the balance between microbiota-dependent and direct mechanisms. Discriminative experiments comparing matched unsaturated AOS (UAOS) and saturated AOS (SAOS) fractions under identical antibiotic regimens would test this prediction directly. A recent review also noted that the direct targets of AOS are not fully resolved [11].

Functional evidence for direct barrier protection comes from Caco-2 monolayers. AOS alleviated the increase in permeability caused by *Clostridioides difficile* toxin TcdA, prevented occludin mislocalization, and accelerated tight junction reassembly [34]. This protection does not depend on microbial metabolism.

#### 3.2.2. Cationic COS via Luminal Interactions

COS carry a positive charge through protonated amino groups (-NH^3+^) with a pKa around 6.3 to 6.5 [39], and the degree of deacetylation determines their charge density. Physicochemical studies showed that the oil-binding capacity of chitosan depends mainly on the degree of deacetylation rather than the degree of polymerization [40]. Unlike AOS, COS act primarily inside the intestinal lumen: they bind bile acids and emulsified lipids, reduce micelle formation, and decrease cholesterol absorption [41,42]. Human evidence for COS on lipid outcomes is limited and inconsistent. A small study in healthy men reported that low-molecular-weight COS (1 g/day, 6 weeks) significantly reduced total cholesterol and LDL cholesterol, but the study had no placebo control [43]. A recent randomized double-blind placebo-controlled trial in 60 overweight and obese women found no significant improvement in lipid profiles after 60 days of COS at 1.5 or 3 g/day [44]. In vitro, COS suppressed adipogenesis and lipid accumulation in 3T3-L1 preadipocytes through downregulation of peroxisome proliferator-activated receptor gamma (PPARγ) and CCAAT/enhancer-binding protein alpha (C/EBPα) and activation of AMP-activated protein kinase (AMPK)-related pathways [45]. These results suggest that the lipid-lowering effect of COS is modest and that its primary site of action is the intestinal lumen.

### 3.3. Structure–Function Parameters

For AOS, the key structural parameters, including the DP, the monomer composition (M/G ratio), the terminal structure, charge, and derivatization, influence its function (listed in Table 1). DP influences bioactivity non-uniformly: DP 2–4 favors anti-inflammatory effects, DP 5 enhances anti-tumor activity, and DP 7–8 optimizes immunomodulation [9]. For GOS, higher DP correlates with stronger anti-inflammatory activity through TLR4/NF-κB inhibition [46].

In addition, among the oligosaccharides examined here, AOS is the only class characterized by a variable mannuronic acid/guluronic acid (M/G) ratio; G-rich sequences enhance Ca^2+^ chelation and antibacterial effects [33], while M-rich sequences influence receptor recognition [9]. The G-block content also governs the physical behavior of AOS in the gastrointestinal tract; anionic alginate polysaccharides inhibit lipid digestion in vitro through calcium chelation and depletion flocculation, and the same ionic interactions underlie the egg-box gelation of G-rich chains [47]. G-rich fragments are preferentially utilized by specific *Bacteroides* strains [48], indicating that monomer composition translates into distinct fermentation outcomes.

**Table 1:** Major bioactivities of AOS with different structural parameters.

Property	Variant/Range	Beneficial Functions	Refs.
DP/MW	DP 2–4	Anti-inflammatory: lowers NO, IL-1β and TNF-α, increases IL-10; oligomannuronate (MAOS) works better against DSS-induced colitis in mice.	[8,49]
	DP 2–4	Anti-obesity: promotes brown fat thermogenesis and improves glucose tolerance.	[38,50]
	DP 5	Anti-tumor: stops tumor cell growth and induces apoptosis.	[8,51]
	DP 7–8	Immunomodulatory: activity changes with DP.	[8]
	Low MW	AOS form dimers during gut fermentation, which slows their degradation.	[52]
	Oligomer vs. polymer	Oligosaccharides are more active than the original alginate polymer; lower MW is important for bioactivity.	[8,50]
M/G ratio	G-rich	Egg-box structure helps Ca^2+^ binding and makes the chain more rigid. Antibacterial activity comes from electrostatic effects shared by G and M blocks.	[8,53]
	G-rich	G-rich fragments are used by gut bacteria like *Bacteroides*, helping shape the microbial community.	[48]
	M-rich	Unsaturated M-type oligosaccharides show neuroprotective effects.	[54]
Terminal structure	UAOS	Enzymatically produced UAOS generally work better than SAOS for anti-obesity and metabolic regulation.	[8,38,55]
	UAOS	UAOS induce alginate utilization genes in gut *Bacteroides*, helping the bacteria break down AOS.	[56,57]
	SAOS	Usually less active than UAOS; can be made by chemo-enzymatic methods.	[8,55,58]
Charge	Negative	Negative charge lets AOS bind to bacterial membranes, block LPS binding to the cell surface, and protect the gut barrier.	[51,59]
Derivatization	Sulfation	Sulfation changes bioactivity: PSS lowers blood lipids and has anti-clotting effects.	[60,61]
	Amidation	Amidation changes how AOS interact with proteins and represents a new way to modify AOS.	[62]

Terminal structure adds another layer: enzymatically produced UAOS show superior antioxidant and anti-obesity activities compared to saturated AOS (SAOS) from acid hydrolysis. The terminal 4,5-unsaturated uronic acid residue generated by alginate lyases is not merely a chemical marker; it is recognized by gut bacteria, since UAOS specifically induce alginate utilization loci (AUL) in *Bacteroides* species [57], and it underlies the higher reactivity of UAOS in antioxidant assays [55]. Saturated AOS with defined structures can now be prepared by chemo-enzymatic routes [58], providing structurally controlled tools for dissecting the contribution of terminal chemistry. Structurally distinct AOS preparations also differ in fermentation kinetics and SCFA profiles in human fecal cultures [63], indicating that terminal structure, like DP and M/G composition, feeds directly into the microbiota-dependent pathway, as discussed in Section 5.

## 4. Direct Mechanisms of AOS in Lipid Metabolism

What distinguishes AOS from neutral prebiotics is their reported capacity to regulate lipid metabolism through host–cell interactions that do not require the microbiota, in addition to the fermentation-dependent actions shared with conventional prebiotics, which will be discussed in Section 5. Here, “direct” denotes cellular actions that do not require microbial metabolism. The direct evidence differs in strength by target tissue: actions at the intestinal mucosal interface, such as tight-junction protection and epithelial signaling, are consistent with an oral route of exposure, whereas direct modulation of the liver, adipose tissue, and vasculature has been demonstrated mainly in vitro or after parenteral administration, and whether oral intake attains sufficient systemic exposure for such effects is not established.

**Figure 2:**
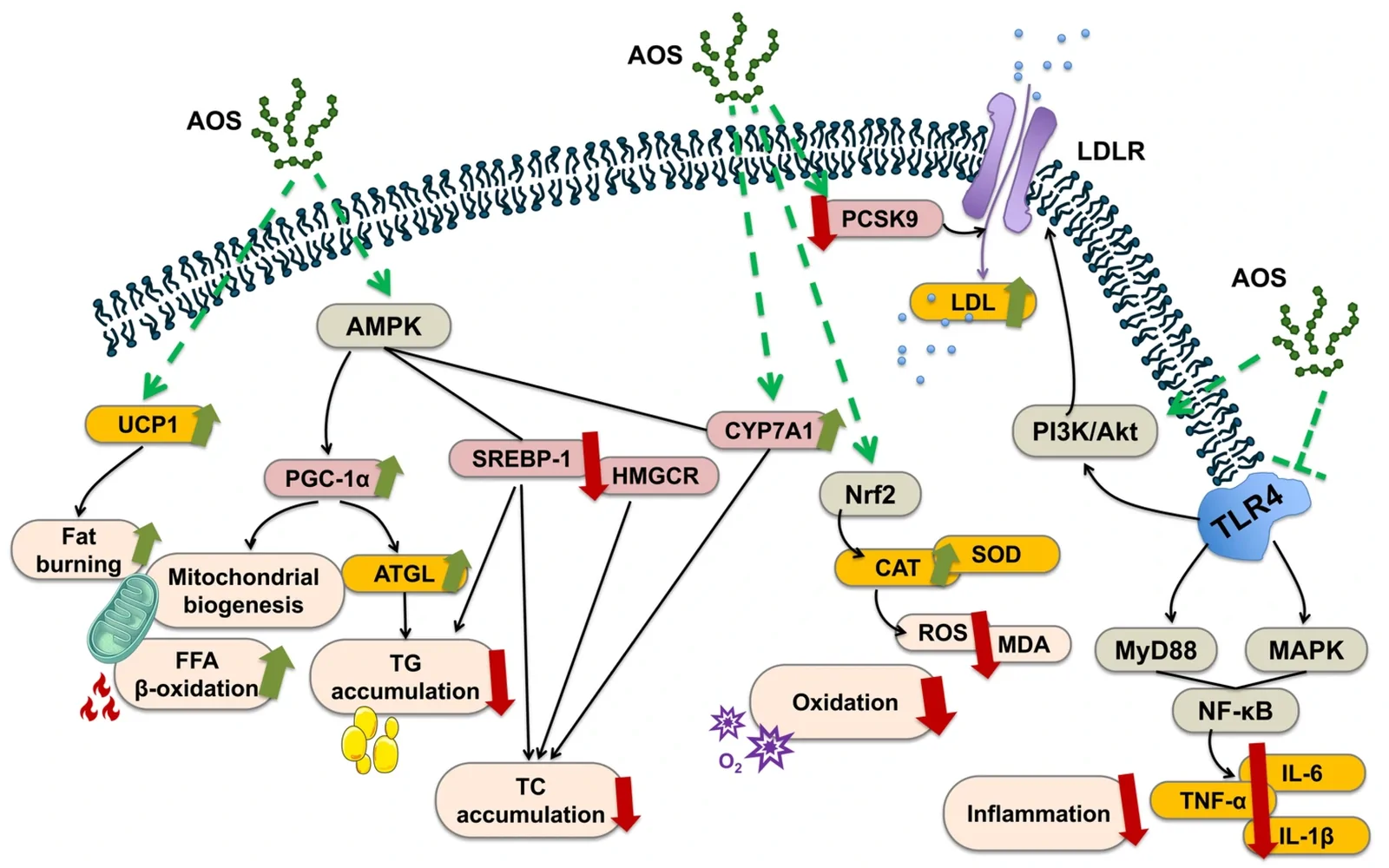
Direct (microbiota-independent) mechanisms of AOS in lipid metabolism at the cellular level. “↑” indicates increasing, upregulating, promoting, accelerating, activating, or maintaining effects of AOS, while T-bar and “↓” indicate decreasing, downregulating, inhibiting, or suppressing effects.

Although pharmacokinetic data are scarce, Nishikawa et al. reported that enzymatically derived unsaturated AOS of DP 2–4 (molecular weight 352–704 Da), given orally to fasted mice, appeared rapidly in portal plasma (peak approximately 24.5 μg/mL at 5 min, about 0.49% of the dose), were cleared within 2 h, and were recovered largely unchanged in urine (peak 425.5 μg/mL at 30 min, still detectable at 6 h) [64]. Systemic exposure to intact AOS thus appears transient and quantitatively limited, and whether it reaches concentrations sufficient for the hepatic and adipose mechanisms discussed below remains unverified; human pharmacokinetic data are lacking. Figure 2 summarizes the direct mechanisms discussed below that do not require the microbiota, as presented at the cellular level.

Xu et al. demonstrated that orally administered AOS retained their anti-obesity and metabolic benefits in high-fat diet-fed mice even when the gut microbiota was depleted with broad-spectrum antibiotics; treated mice showed reduced body weight, improved hepatic lipid metabolism, and enhanced brown adipose tissue (BAT) thermogenesis [38]. This result indicates that at least part of the oral efficacy of the tested preparation is microbiota-independent, although the same study also reported reduced small-intestinal lipid absorption, and the site of this microbiota-independent action (systemic delivery versus luminal or mucosal effects) cannot be distinguished without tissue-exposure data. Liang et al. reported that orally administered AOS protected against renal ischemia–reperfusion injury in rats through pathways at least partially dependent on MRC1 [10]; whether this reflects direct interaction of intact AOS with MRC1-expressing tissues after oral intake or is mediated indirectly remains unresolved. Intraperitoneal AOS administration, which bypasses the gastrointestinal tract, also ameliorated metabolic disorders [38]. Such parenteral data serve as mechanistic proof-of-concept: they demonstrate direct activity of AOS when systemic delivery is achieved, but they do not by themselves establish efficacy after oral intake. In vitro studies on hepatocytes and adipocytes, detailed in Section 4.1 and Section 4.2, likewise support direct, microbiota-independent modulation of lipid metabolism [65].

### 4.1. Liver: AMPK/PGC-1α Pathway, HMGCR Inhibition, and Lipophagy

In hepatocytes, AOS directly activate AMP-activated protein kinase (AMPK), promoting lipophagy and peroxisome proliferator-activated receptor γ coactivator 1α (PGC-1α)-dependent fatty acid oxidation [65]. PGC-1α inhibition reversed AOS-mediated lipid reduction, supporting pathway causality. Zhuang et al. reported that AOS suppress 3-hydroxy-3-methylglutaryl-CoA reductase (HMGCR) promoter activity in a dose-dependent manner in oleic acid-induced steatosis models [66]. In HepG2 cells, low-molecular-weight sulfated guluronic acid oligosaccharides activate AMPK, inhibit HMGCR, downregulate sterol regulatory element-binding protein 1 (SREBP-1), and upregulate cholesterol 7α-hydroxylase (CYP7A1) and low-density lipoprotein receptor (LDLR) expression [67]. AOS further enhance LDL uptake by upregulating LDLR and suppressing proprotein convertase subtilisin/kexin type 9 (PCSK9), with LDLR upregulation involving PI3K/Akt-mediated sterol regulatory element-binding protein 2 (SREBP-2) activation [68]. These hepatocellular effects are direct and do not require microbial metabolites.

### 4.2. Adipose Tissue: UCP1 Upregulation and Mitochondrial Biogenesis

AOS promote brown adipose tissue activation, as evidenced by upregulated uncoupling protein 1 (UCP1) expression, increased mitochondrial DNA copy number, and enhanced fatty acid oxidation gene expression following oral AOS administration [38]. In 3T3-L1 adipocytes, the oligomannuronate-chromium(III) complex (OM2) reduces triglyceride accumulation via the AMPK-PGC-1α pathway, promoting mitochondrial biogenesis, enhancing fatty acid oxidation and lipolysis, and increasing the expression and promoter activity of adipose triglyceride lipase (ATGL); because chromium(III) is itself an AMPK activator, the contribution of the oligosaccharide component alone cannot be isolated from this study, and the direct anti-adipogenic evidence for pure AOS comes from enzymatically prepared AOS [69]. Enzymatically prepared AOS directly inhibit lipid accumulation in differentiated 3T3-L1 adipocytes, whereas the parent alginate polymer does not, underscoring the importance of molecular weight reduction for direct cellular activity [70].

### 4.3. Anti-Inflammatory and Antioxidant Actions for Lipid Regulation

AOS have been reported to activate the nuclear factor erythroid 2-related factor 2 (Nrf2) antioxidant pathway via p62/SQSTM1-Keap1 dissociation, upregulating superoxide dismutase (SOD), catalase (CAT), and glutathione peroxidase while reducing reactive oxygen species (ROS) and malondialdehyde (MDA) levels. These observations were made in cultured gastric epithelial cells and in animal studies, in which AOS were administered alone or in combination with other ingredients [71,72]. Beyond direct effects on hepatocytes and adipocytes, AOS also suppress oxidative stress in vascular endothelial cells through integrin-α/focal adhesion kinase (FAK)/phosphoinositide 3-kinase (PI3K) signaling [73], and in gastric epithelial cells through p62-Keap1-Nrf2 activation. These findings indicate that the antioxidant actions of AOS extend across multiple tissues [72]. Inflammatory signaling is suppressed through inhibition of TLR4/MAPK/NF-κB and NOD-like receptor family pyrin domain-containing 3 (NLRP3) inflammasome activation, reducing tumor necrosis factor-alpha (TNF-α), interleukin-1 beta (IL-1β), and interleukin-6 (IL-6) [74,75]. These effects have been demonstrated directly in cultured macrophages, hepatocytes, and intestinal epithelial cells.

### 4.4. Receptor-Mediated Effects via MRC1 and TLR4 Signaling Pathways

A single recent study has proposed MRC1 as a candidate receptor for AOS. In molecular docking predictions, AOS are predicted to bind to MRC1 via hydrogen bonding with residues including E725, N727, E733, T743, S745, and N747, and in MRC1-knockout mice, the protective effect was significantly weakened [10]. AOS also block LPS binding to the cell surface and attenuate TLR4/MyD88/NF-κB signaling [59], and engage integrin-α receptors, activating downstream FAK/PI3K signaling in endothelial cells [73]. Together, these observations point to candidate receptor-mediated pathways through which AOS may act independently of the microbiota, and distinguish them from neutral oligosaccharides for which no comparable direct receptor interactions, as far as we know, have been reported.

Support for microbiota-independent actions of AOS rests on several independent lines of evidence, such as microbiota-depleted animal models, intraperitoneal administration bypassing the gut, and in vitro studies on isolated hepatocytes and adipocytes [38,65,67,70]. The proposed involvement of MRC1 raises the possibility of direct cellular effects, although this remains tentative [10]. However, several limitations remain. One important issue is that the MRC1 evidence rests on a single study combining molecular docking with an oral-dosing rat model of renal ischemia–reperfusion injury and MRC1-knockout verification in mice [10]; MRC1’s canonical carbohydrate ligands are mannose-, fucose-, and GlcNAc-terminated glycans recognized through Ca^2+^-dependent coordination [76], whereas AOS carry uronic acid residues whose predicted binding mode is non-canonical, and the knockout phenotype may partly reflect impaired macrophage clearance. Moreover, MRC1 is expressed mainly on macrophages and hepatic sinusoidal endothelial cells rather than on the apical surface of the intestinal epithelium, so an orally administered AOS would need to reach the systemic circulation to interact with this receptor; systemic exposure after oral intake has not been quantified. Yet, the majority of evidence comes from rodent models and cell lines; no human clinical trial has yet examined AOS for lipid metabolism outcomes. The AMPK activation data are predominantly from in vitro studies at concentrations that may not be achievable through oral administration, given that AOS are hydrophilic oligosaccharides whose oral bioavailability has not been characterized. Besides, the relative contribution of direct versus microbiota-mediated pathways under physiological conditions (i.e., when the gut microbiota is intact) has not been quantified.

## 5. Gut-Dependent Mechanisms of AOS in Lipid Metabolism

**Figure 3:**
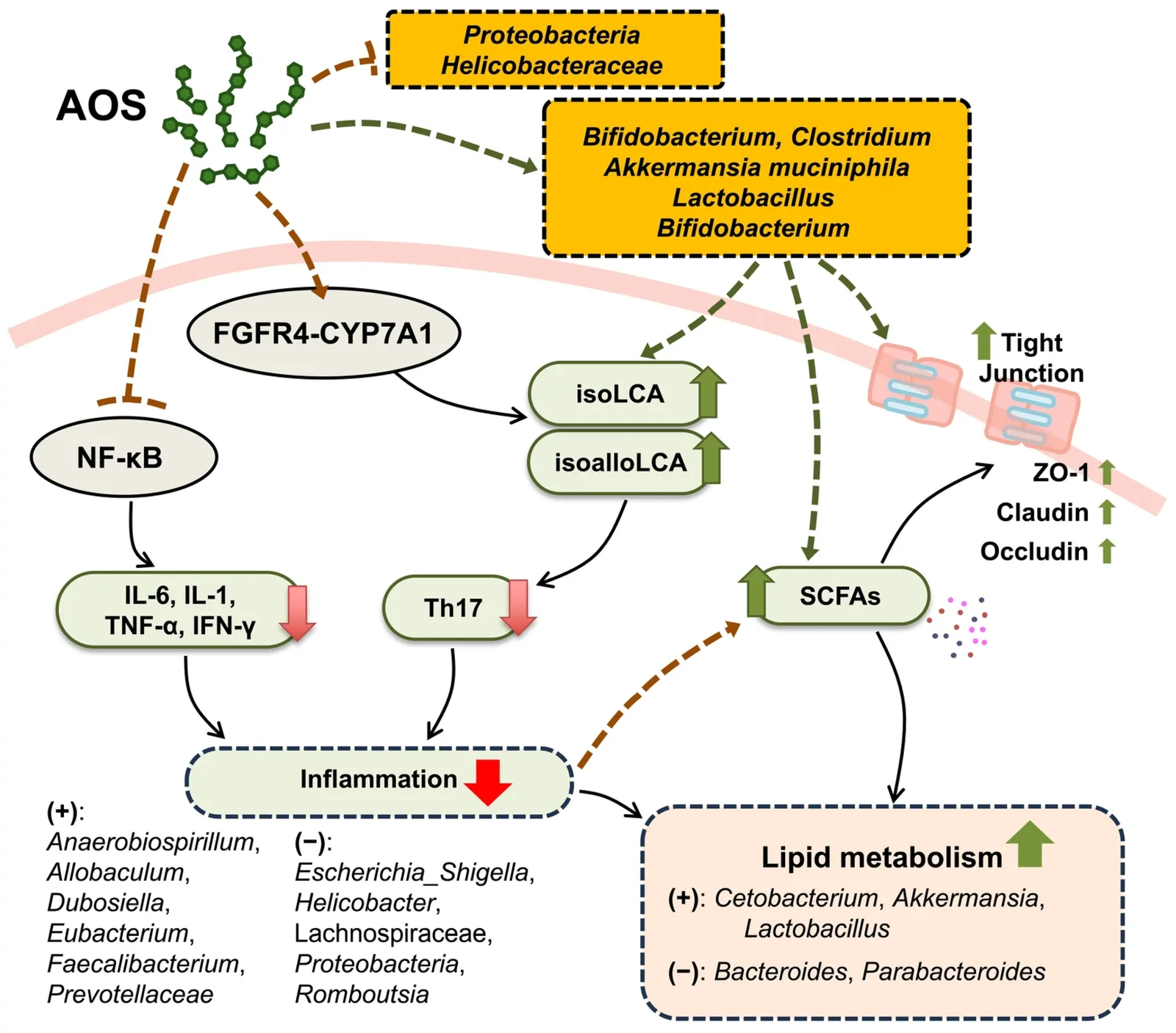
Gut-dependent mechanisms of AOS in lipid metabolism. AOS remodel the gut microbiota, promote SCFA production, strengthen intestinal barrier integrity, and modulate bile acid metabolism (isoLCA/isoalloLCA; FXR–FGFR4–CYP7A1), with downstream effects on mucosal immunity and systemic lipid metabolism. “↑” indicates increasing, upregulating, promoting, accelerating, activating, or maintaining effects of AOS, while T-bar and “↓” indicate decreasing, downregulating, inhibiting, or suppressing effects.

In parallel with its direct actions, AOS modulate lipid metabolism through gut modulation (Figure 3), which largely depends on gut-microbiota regulation. AOS selectively enrich beneficial bacteria including *Akkermansia muciniphila*, *lactobacilli*, and *Bifidobacterium* while suppressing *Proteobacteria* and *Helicobacter* [12,66,77]. The resulting SCFAs, including acetate, propionate, and butyrate, activate GPR41/GPR43 and inhibit HDACs, improving insulin sensitivity and reducing hepatic lipogenesis [4]. Fecal microbiota transplantation experiments in AOS-treated models have reproduced functional benefits in recipient mice, including improved hepatic and circulating lipid profiles in high-fat diet-fed recipients, supporting a causative role of the microbiota in those models [78].

Beyond compositional shifts, recent studies have elucidated the molecular basis of AOS utilization by gut bacteria at the gene cluster level. The human gut bacterium *Bacteroides ovatus* harbors a dedicated polysaccharide utilization locus (PUL) encoding three alginate lyases that collaboratively degrade AOS [56]. A subsequent study demonstrated that UAOS specifically induce the expression of alginate utilization loci in *Bacteroides* species, revealing that the terminal unsaturation of enzymatically produced AOS influences bacterial recognition and uptake [57]. *Bacteroides xylanisolvens* has been identified as a keystone species responsible for AOS fermentation, with lower-MW AOS (0.8 kDa) producing higher acetate and butyrate yields than higher-MW fractions [79]. The M/G ratio also dictates selectivity: G-rich fragments are preferentially utilized by certain *Bacteroides* strains, suggesting that AOS structural tuning can shape the resulting microbial community structure [48].

### 5.1. Bile Acid Metabolism and Gut Barrier

AOS can correct bile acid dysmetabolism in estrogen-deficient mice by increasing isolithocholic acid (isoLCA) and isoallolithocholic acid (isoalloLCA) levels [80] (Figure 3). They have also been found to upregulate tight junction proteins, e.g., occludin, claudin, and zonula occludens-1 (ZO-1), increase mucus thickness, and reduce intestinal permeability through TLR4/NF-κB pathway inhibition [59,81]. In addition, Cha et al. reported that UAOS activate intestinal farnesoid X receptor (FXR)-fibroblast growth factor 15 (FGF15) signaling, suppress hepatic CYP7A1, and reduce bile acid synthesis in non-obese NAFLD mice, with effects associated with *Akkermansia* and *Lachnoclostridium* enrichment [82]. These effects reduce endotoxin translocation and systemic inflammation, indirectly improving lipid metabolism. Moreover, alginate-encapsulated postbiotics alleviated dextran sulfate sodium (DSS)-induced colitis by modulating the gut microbiota, further supporting the barrier-protective potential of alginate-based formulations [5].

### 5.2. Methodological Considerations in Gut Microbiota Studies

Current research progress on the gut-dependent lipid-regulating effects of AOS is summarized in Table 2. Several methodological factors should be considered when interpreting the gut microbiota findings reviewed above. Most studies used 16S rRNA amplicon sequencing, which typically provides taxonomic resolution at the genus level and cannot reliably resolve species or strain-level variation. Metagenomic or metabolomic approaches would better characterize the functional capacity of the AOS-modulated microbiota. The AOS dosage, molecular weight distribution, and M/G composition vary considerably across studies, which makes direct comparisons difficult. Fecal microbiota transplantation experiments [78,83] provide strong evidence for a causal role of the microbiota, but such experiments cannot distinguish between the effects of live bacteria, bacterial metabolites, and structural components. Standardization of AOS preparations and experimental protocols across studies would greatly strengthen the evidence base. In vitro simulated digestion and fermentation using human gut microbiota offer a controlled model for such studies [63].

An additional consideration concerns the translational gap between the rodent models that dominate this literature and human physiology. Most of the gut-dependent effects reviewed above were demonstrated in mice, and species differences in cholesterol metabolism limit direct extrapolation: mice lack cholesteryl ester transfer protein (CETP), carry most of their plasma cholesterol in high-density lipoproteins, and synthesize a substantial fraction of cholesterol in the intestine, whereas in humans low-density lipoproteins dominate and cholesterol synthesis is predominantly hepatic [84]. Intestinal lumen-facing actions of AOS (e.g., effects on cholesterol absorption or bile acid handling) may therefore carry more weight in mice, and their relative contribution cannot be assumed to translate to humans. Clinical data that would resolve this uncertainty are lacking for both fermentation-dependent and direct mechanisms of AOS.

**Table 2:** Studies on the lipid-regulating effects of AOS mediated by the gut microbiota.

Model/System	Key Findings	Key Taxa Modulated	Refs.
HFD-fed mice	Enriched *Akkermansia muciniphila*, *lactobacilli*, *Bifidobacterium*; suppressed Proteobacteria and Helicobacter	*Akkermansia*, *lactobacilli*, *Bifidobacterium*	[11,12,85]
HFD-fed mice	Reduced TG and LDL-C, inhibited lipogenic gene expression, and enhanced SCFA production	*Akkermansia muciniphila*, *L. reuteri*, *L. gasseri*	[86]
HFD-fed mice	Unsaturated AOS attenuated obesity-related metabolic abnormalities; effects reversed by antibiotics	Overall community (antibiotic-reversible)	[12]
HFD-fed mice (FMT recipients)	FMT from AOS-dosed donor mice reduced blood TG, TC, and liver lipid content, with increased blood n-3 PUFA	*Bacteroides*, *Mucispirillum*	[78]
Non-obese NAFLD mice	UAOS activated intestinal FXR-FGF15 signaling and suppressed hepatic CYP7A1, reducing bile acid synthesis	*Akkermansia*, *Lachnoclostridium*	[82]
Estrogen-deficient mice	Corrected bile acid dysmetabolism by increasing isoLCA and isoalloLCA levels	Not reported	[80]
Type 2 diabetic mice and NAFLD-patient fecal microbiota	Improved glycolipid metabolism and SCFA production in diabetic mice; in vitro fermentation by NAFLD-patient microbiota promoted beneficial bacteria	*lactobacilli*	[66]
Low-birth-weight piglets	Improved hepatic glucose and lipid metabolism through gut microbiota modulation	Not reported	[87]
Intestinal epithelium and mice	Upregulated tight junction proteins and mucus; reduced permeability via TLR4/NF-κB inhibition	Not reported	[59,81]
*Bacteroides ovatus*	A dedicated polysaccharide utilization locus with three alginate lyases degrades AOS	*Bacteroides ovatus*	[56]
*Bacteroides* species	UAOS induced alginate utilization loci; terminal unsaturation influenced recognition and uptake	*Bacteroides clarus*	[57]
Fecal fermentation	*Bacteroides xylanisolvens* as keystone species; 0.8 kDa AOS yielded higher acetate and butyrate	*Bacteroides xylanisolvens*	[79]
Fecal fermentation	G-rich fragments preferentially utilized by specific *Bacteroides* strains	*Bacteroides* (specific strains)	[48]
Antibiotic-depleted mice	Anti-obesity effects retained after microbiota depletion, indicating coexistence of direct mechanisms	Microbiota depleted (control)	[38]

## 6. AOS Industry Development, Regulatory Landscape, and Challenges

### 6.1. Global Market Overview and Development History

Market size estimates for AOS vary widely across market research firms, from tens of millions to hundreds of millions of US dollars, and the individual estimates cannot be attributed to published reports. This spread is not merely a statistical artifact; it reflects the absence of an agreed definition of AOS as a product category, and the same ambiguity complicates quality control and regulatory classification. AOS represent an emerging segment within the much larger prebiotics market, and they compete with mature products such as FOS and GOS on the basis of multifunctional bioactivity rather than price. The Asia-Pacific region accounts for the largest share of the AOS market, and food and beverage applications represent the largest end-use segment, consistent with China’s dominant position in seaweed harvesting and alginate production.

The development of AOS spans several decades. Early work established enzymatic and chemical methods for degrading alginate into oligosaccharides, and studies from the 2010s onward revealed a broad spectrum of biological activities, including anti-inflammatory, antioxidant, and lipid-regulating effects. On the pharmaceutical side, GV-971 (sodium oligomannate), an M-type AOS developed for Alzheimer’s disease, received conditional approval from China’s National Medical Products Administration (NMPA) in November 2019 and was listed in the national medical insurance scheme at the end of 2021 (effective January 2022), showing both the promise and the difficulties of marine oligosaccharide drug development. In the same therapeutic direction, the alginate oligosaccharide drug candidate OligoG completed early clinical trials for cystic fibrosis, but its Phase IIb trial (NCT03822455) was terminated early, and the reasons for termination have not been publicly disclosed, underscoring the challenges of translating AOS into pharmaceuticals. In the food sector, an industry value chain is now taking shape. China is the world’s largest producer of farmed seaweeds, and the upstream alginate industry is anchored by producers, while the downstream AOS segment is led by a series of Chinese companies located in Qingdao, which have invested in dedicated food-grade AOS production lines. Internationally, alginate production is concentrated in a few seaweed-rich regions, with Norway, France, and Chile among the major producers alongside China. European public funding supported the early clinical development of OligoG. Moreover, Japan approved low-molecular-weight sodium alginate as a food for specified health uses more than a decade ago. Hence, the field is advancing on a global scale, from raw-material production to clinical development.

### 6.2. Regulatory Approvals

A decisive milestone was reached in February 2026, when China’s National Health Commission (NHC) approved alginate oligosaccharides (named brown algae oligosaccharides in the announcement) as a novel food raw material, among 22 substances covered by Announcement No. 1 of 2026. The approval specifies a recommended daily intake of no more than 4 g, based on an oligosaccharide content of 90.0 g/100 g, and states that the ingredient should not be consumed by infants, young children, or pregnant and lactating women. The announced production process starts from sodium alginate of brown seaweeds such as Laminaria japonica, Sargassum, Macrocystis, and Ascophyllum, which is dissolved in hydrochloric acid and degraded at high temperature to yield oligosaccharides with a degree of polymerization of 2 to 10. This approval closed a long-standing regulatory gap that limited the food use of AOS in China. Elsewhere, regulatory status is uneven and reflects different institutional logics. In Japan, low-molecular-weight sodium alginate has been approved as a Food for Specified Health Uses (FOSHU) ingredient (for example, the Takeda product Healthy-In W^®^, licensed in 2018). This is a product-level authorization that attaches health claims to individual products, not to the ingredient class. In the United States, sodium alginate is generally recognized as safe (GRAS; 21 CFR 184.1724), but AOS have no independent GRAS notification or new dietary ingredient filing, and in the European Union AOS have not been authorized as a novel food under the centralized procedure. Registered clinical development of AOS-based products remains limited to seven registered trials of OligoG in cystic fibrosis, five of which have been completed (ClinicalTrials.gov, searched July 2026 using the term “OligoG”; including NCT03822455). China has moved first in part because its ingredient-level authorization covers the ingredient class as a whole; the pace of commercialization elsewhere will depend on how quickly evidence meets the evidentiary standards of each system.

The production process specified in the Chinese approval (acid hydrolysis at high temperature) yields predominantly saturated AOS (SAOS, DP 2–10), whereas the most active preparations in the mechanistic literature reviewed here are enzymatically produced UAOS. This divergence between the approved product structure and the research-optimized candidate structure has direct implications for clinical translation: future human trials should specify which AOS structure is being tested, and bridging studies are needed to determine whether the regulatory product retains the bioactivities attributed to UAOS in preclinical models.

### 6.3. Challenges

Applications of AOS span food ingredients and dietary supplements, as discussed in the preceding sections; agricultural applications are an emerging direction beyond the scope of this review. In agriculture, for example, AOS prime biocontrol yeasts such as *Debaryomyces hansenii* against postharvest pathogens [88,89]. The obstacles to wider commercialization form a causal chain rather than a list of independent problems. It should be noted that AOS are produced as mixtures with variable degree of polymerization and M/G ratio; without standardized reference materials and analytical methods, batch-to-batch consistency cannot be guaranteed; without consistent material, clinical studies cannot be reproduced; and without reproducible evidence, regulatory health claims cannot be obtained. Besides, consumer awareness is a further constraint that the 2026 Chinese approval and the ongoing expansion of production capacity have only begun to address. Breaking this chain at its first link, by establishing pharmacopoeia-grade AOS reference standards, would be the most important step forward.

## 7. Conclusions and Future Perspectives

Overall, the evidence reviewed here suggests that charge polarity is one of several structural parameters, alongside monomer composition, glycosidic linkage, and molecular weight, that collectively influence oligosaccharide mechanisms. The charge-based framework proposed here offers a useful heuristic for generating testable hypotheses, although confirmatory studies with structurally matched, charge-only oligosaccharide pairs are needed to establish causality. Neutral species act predominantly through fermentation, whereas charged species additionally engage host cells through electrostatic interactions, and charge polarity offers a practical framework for predicting the mode of action. AOS are the most thoroughly characterized charged prebiotics, operating through both microbiota-dependent fermentation and direct host–cell interactions that converge on lipid metabolism. The most pressing gaps are clinical rather than mechanistic: no clinical trial has yet tested AOS for lipid outcomes, and the pharmacokinetics of orally administered AOS remain uncharacterized.

It is noted that the oligosaccharide families discussed here differ not only in charge but also in monomer composition, glycosidic linkage, molecular weight, and biological source. Charge polarity therefore cannot be experimentally separated from backbone structure in the studies reviewed here. Direct evidence from structurally matched pairs that differ only in charge remains limited; nonetheless, studies in other glycan systems show that charge modification alone can alter receptor engagement, supporting the plausibility of charge as a contributing parameter [90].

In terms of AOS, future work should prioritize the following three directions: First, in production, enzymatic and membrane-based processes that deliver structurally defined AOS fractions (defined DP, M/G ratio, and terminal unsaturation), together with pharmacopoeia-grade reference standards, would shift the field from mixture-based empiricism to a structure-activity-driven paradigm. Second, in clinical evidence, trials in metabolically at-risk populations using defined AOS fractions and lipid-related endpoints should test whether rodent findings translate to humans. Pharmacokinetic studies should establish whether oral AOS reaches systemic circulation in active concentrations or acts predominantly at the intestinal interface. Third, in application, formulation strategies that exploit alginate’s own encapsulation properties, combinations with probiotics or postbiotics, and stratification of responders by microbiota or genetic background could turn the charge-based framework into a tool for personalized prebiotic design.

## Data Availability

No new data were created or analyzed in this study. Data sharing is not applicable to this article.

## Abbreviations

The following abbreviations are used in this manuscript:

AMPK	AMP-activated protein kinase
AOS	Alginate oligosaccharides
AUL	Alginate utilization loci
BAT	Brown adipose tissue
C/EBPα	CCAAT/enhancer-binding protein alpha
CAT	Catalase
COS	Chitosan oligosaccharides
CYP7A1	Cholesterol 7α-hydroxylase
DP	Degree of polymerization
DSS	Dextran sulfate sodium
ERK1/2	Extracellular signal-regulated kinases 1 and 2
FFAR2/3	Free fatty acid receptors 2 and 3
FGF15	Fibroblast growth factor 15
FMT	Fecal microbiota transplantation
FOS	Fructo-oligosaccharides
FOSHU	Food for Specified Health Uses
FXR	Farnesoid X receptor
GOS	Galacto-oligosaccharides
GPR41/43	G protein-coupled receptors 41 and 43
GRAS	Generally Recognized as Safe
HDAC	Histone deacetylase
HMGCR	HMG-CoA reductase
IL-10	Interleukin-10
IL-1β	Interleukin-1 beta
IL-22	Interleukin-22
IL-6	Interleukin-6
LDL	Low-density lipoprotein
LDL-C	Low-density lipoprotein cholesterol
LDLR	Low-density lipoprotein receptor
LPS	Lipopolysaccharide
MAOS	Mannuronate oligosaccharides
MAPK	Mitogen-activated protein kinase
MASLD	Metabolic dysfunction-associated steatotic liver disease
MDA	Malondialdehyde
MOS	Manno-oligosaccharides
MRC1	Mannose receptor C-type 1 (CD206)
MyD88	Myeloid differentiation primary response 88
NAFLD	Non-alcoholic fatty liver disease
NF-κB	Nuclear factor kappa B
NHC	National Health Commission
NLRP3	NOD-like receptor family pyrin domain-containing 3
NMPA	National Medical Products Administration
Nrf2	Nuclear factor erythroid 2-related factor 2
OM2	Oligomannuronate-chromium(III) complex
PCSK9	Proprotein convertase subtilisin/kexin type 9
PGC-1α	Peroxisome proliferator-activated receptor gamma coactivator 1-alpha
PPARγ	Peroxisome proliferator-activated receptor gamma
PSS	Propylene glycol alginate sodium sulfate
PUL	Polysaccharide utilization loci
ROS	Reactive oxygen species
SAOS	Saturated alginate oligosaccharides
SCFA	Short-chain fatty acid
SOD	Superoxide dismutase
SREBP-1	Sterol regulatory element-binding protein 1
TLR4	Toll-like receptor 4
TNF-α	Tumor necrosis factor alpha
UAOS	Unsaturated alginate oligosaccharides
UCP1	Uncoupling protein 1
XOS	Xylo-oligosaccharides
ZO-1	Zonula occludens-1
